# Effects of high-intensity statin combined with telmisartan versus amlodipine on glucose metabolism in hypertensive atherosclerotic cardiovascular disease patients with impaired fasting glucose: A randomized multicenter trial

**DOI:** 10.1097/MD.0000000000030496

**Published:** 2022-09-09

**Authors:** Chan Joo Lee, Jung-Hoon Sung, Tae-Soo Kang, Sungha Park, Sang-Hak Lee, Jong-Youn Kim, Byeong-Kuek Kim

**Affiliations:** a Division of Cardiology, Severance Cardiovascular Hospital, Yonsei University College of Medicine, Seoul, Republic of Korea; b Department of Cardiology, CHA Bundang Medical Center, CHA University, Seongnam, Gyeonggi-do, Republic of Korea; c Division of Cardiology, Department of Internal Medicine, Dankook University College of Medicine, Cheonan, Republic of Korea; d Division of Cardiology, Heart Center, Gangnam Severance Hospital, Yonsei University College of Medicine, Seoul, Republic of Korea.

**Keywords:** angiotensin-II-receptor blockers, calcium channel blocker, diabetes mellitus, impaired fasting glucose, insulin resistance, statin

## Abstract

There is lacking evidence that telmisartan can improve insulin resistance in patients on high-intensity statins. This study compared the effects of telmisartan and amlodipine on glucose metabolism in hypertensive atherosclerotic cardiovascular disease (ASCVD) patients with impaired fasting glucose (IFG) requiring high-intensity rosuvastatin therapy.

**Methods:**

Ninety-nine patients were randomly assigned to 2 groups [telmisartan-statin group (n=48) and amlodipine-statin group (n=51)] as add-on therapy to high-intensity rosuvastatin therapy (20 mg). The primary endpoint was to assess insulin resistance using the homeostatic model assessment (HOMA-IR) value at week 24. The secondary endpoint was the change in glucose metabolism indices from baseline to week 24.

**Results:**

The HOMA-IR at week 24 (2.4 [interquartile range, 1.8–3.8] versus 2.7 [1.7–3.7]; *P* = .809) and changes in the HOMA-IR from baseline to week 24 (−7.0 [−29.0 to 21.0] versus −5.5 [−53.3 to 27.3]; *P* = .539) were not significantly different between 2 groups. However, the fasting glucose level at week 24 was significantly lower in the telmisartan-statin group than in the amlodipine-statin group (107.7 ± 13.4 mg/dL versus 113.3 ± 12.4 mg/dL; *P* = .039) and significantly decreased in the telmisartan-statin group (−3.2 ± 8.6% versus 3.8 ± 13.2%; *P* = .003). The proportion of patients with fasting glucose ≥100 mg/dL (71.1% versus 89.6%; *P* = .047) or new-onset diabetes mellitus (12.5% versus 31.4%, *P* = .044) at week 24 was also significantly lower in the telmisartan-statin group than in the amlodipine-statin group.

**Conclusion:**

In comparison to amlodipine, telmisartan did not decrease the HOMA-IR. However, telmisartan preserved insulin secretion, led to a regression from IFG to euglycemia and prevented new-onset diabetes mellitus in ASCVD patients with IFG requiring high-intensity statins.

## 1. Introduction

For prevention of high cardiovascular risk of atherosclerotic cardiovascular disease (ASCVD), strict control of low-density lipoprotein-cholesterol (LDL-C) using statins is recommended.^[1]^ Despite several beneficial effects of statin therapy, statin use is associated with an increased risk of developing diabetes mellitus (DM).^[2]^ A meta-analysis of randomized clinical trials showed that statin therapy was associated with an increased risk of new-onset DM (NODM).^[3]^ There are several experimental studies that have reported that statins act on beta cells, which reduce insulin secretion and inhibit glucose uptake in adipocytes and skeletal muscles, thus leading to insulin resistance and DM.^[4,5]^ Particularly, these phenomena are more pronounced using high-intensity statins than moderate- or low-intensity statins, and statin-induced NODM is more likely to occur in people with a high risk of diabetes.^[6,7]^ Therefore, in patients with insulin resistance such as impaired fasting glucose (IFG) or elevated hemoglobin A1c (HbA1c), the risk of NODM caused by high-intensity statins may be increased.^[8]^ The use of statins in hypertensive patients increases the risk of NODM, and this risk has a proportional increase as the intensity of statin increases.^[9]^ These patients at risk of NODM, requiring the management of hypertension and LDL-C, could expect a decrease in insulin resistance using appropriate antihypertensive drugs. Among angiotensin-II-receptor blockers (ARB), telmisartan has been found to reduce insulin resistance by activating peroxisome proliferator-activated receptor gamma (PPARγ).^[10]^ Telmisartan showed superior ability to improve insulin resistance induced by rosuvastatin 10 mg compared to other ARBs.^[11]^ However, studies on the effects of telmisartan on changes in glucose metabolism including insulin resistance caused by high-intensity statins are lacking. In addition, telmisartan may be superior in preventing cardiovascular disease compared to amlodipine, a calcium channel blocker, independent of the BP-lowering effect. Telmisartan increases PPAR-r gene expression more than amlodipine, and has a positive influence on glycemic control, including insulin resistance.^[12]^ Prior studies examined the effects of telmisartan in patients with insulin resistance, regardless of the use of statins, and evaluated the changes in metabolic parameters without comparison with amlodipine.^[11,13,14]^ Therefore, the study aimed to compare the effects of telmisartan and amlodipine on glucose metabolism in hypertensive ASCVD patients with IFG requiring high-intensity rosuvastatin therapy.

## 2. Participants and methods

### 2.1. Study design

**Figure 1.**
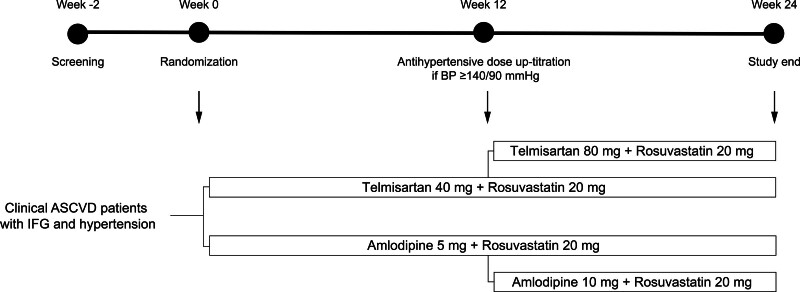
Study design and flow. BP = blood pressure; IFG = impaired fasting glucose.

This trial, the COMPROMISE (effect of high-dose rosuvastatin combined with telmisartan or amlodipine on glucose metabolism in ASCVD patients with impaired fasting glucose and hypertension) trial was a 24-week randomized, open-label, parallel, multicenter trial conducted at 4 sites in South Korea between October 2018 and May 2019. The study design is presented in Figure 1. After randomization, all participants have initially treated with telmisartan 40 mg/rosuvastatin 20 mg once daily or amlodipine 5 mg/rosuvastatin 20 mg once daily for the first 12 weeks. Participants who were previously taking antihypertensive drugs or statins changed their medications without a washout period. Then, all participants were up-titrated to telmisartan 80 mg/rosuvastatin 20 mg once daily or amlodipine 10 mg/rosuvastatin 20 mg once daily for an additional 12 weeks if the participant did not meet blood pressure (BP) goals (i.e., mean sitting systolic BP/diastolic BP of 140/90 mm Hg).^[15]^

### 2.2. Randomization and masking

After screening, participants were randomly assigned to the combination therapy with telmisartan and high-intensity rosuvastatin (telmisartan-statin group) or the combination therapy with amlodipine and high-intensity rosuvastatin (amlodipine-statin group) in a 1:1 ratio. Precoded concealed envelopes generated by permuted-block randomization with a block size of 2 stratified were used to assign groups. The clinical research coordinators in each center performed the randomization.

### 2.3. Ethics

The ethics review boards of the appropriate ethics review boards and each participating hospital approved the study protocol. The study was conducted in accordance with the principles of Declaration of Helsinki, and all patients provided written informed consent.

### 2.4. Study population

Patients aged 19–75 years who met the following conditions were enrolled: patients with clinical ASCVD requiring high-intensity statin therapy (clinical ASCVD included acute coronary syndrome, history of myocardial infarction, stable or unstable angina, history of coronary revascularization, stroke, or transient ischemic attack, peripheral arterial disease, or history of peripheral arterial revascularization)^[1]^; patients who were taking antihypertensive drugs or with a systolic or diastolic BP of >140 mmHg or >90 mmHg; patients who met the criteria for IFG (fasting plasma glucose of 100–125 mg/dL or HbA1c of 5.7–6.4%) and had not been diagnosed with DM before.^[16]^

The exclusion criteria were as follows: patients who were treated for secondary or malignant hypertension; uncontrollable DM with HbA1c ≥10%; total cholesterol of ≥300 mg/dL; fasting LDL-C of ≤70 mg/dL in statin-naïve patients; fasting triglyceride of ≥500 mg/dL; history of muscular disease or rhabdomyolysis owing to statin use; hypersensitive to statins or ARBs (a history of rash or allergic reaction to these drugs in the medical records or the medical examination by interview); contraindications stated in the single-pill combination of telmisartan or rosuvastatin (severe renal disease, aspartate aminotransferase or alanine aminotransferase >3-fold upper normal limit or history of active liver disease, creatine phosphokinase >3-fold upper normal limit, or hyperkalemia); those who were participating in clinical trials of other investigational products; those who could not discontinue all other treatments for hypertension or hyperlipidemia than the clinical trial drugs, concomitant medications, and supplements that can affect the therapeutic effects of hypertension and hyperlipidemia; and if the investigator considers the participant to be ineligible for the trial.

### 2.5. Efficacy assessments

The primary endpoint was the difference in the homeostatic model assessment for insulin resistance (HOMA-IR) at week 24. HOMA-IR was calculated using the formula: fasting insulin (μU/mL) × fasting glucose (mg/dL)/405. The secondary endpoints were the changes from the baseline to week 12 or 24 in the following variables: HOMA-IR, homeostasis model assessment of β-cell function (HOMA-B), fasting blood glucose, fasting insulin, HbA1c, lipid profiles (total cholesterol, triglyceride, LDL-cholesterol, and high-density lipoprotein-cholesterol (HDL-C)); the proportion of participants with fasting plasma glucose of ≥100 mg/dL and NODM at week 24. HOMA-B was calculated using the formula: [fasting insulin (μU/mL) × 360]/ [fasting glucose (mg/dL) – 63]. NODM was defined as the fasting plasma glucose of ≥125 mg/dL or HbA1c of ≥6.5% at week 24.^[16]^ The BP was measured during every visit to the out-patient clinic, and the adverse events, including their severity and causal relationship, were evaluated during the study period.

### 2.6. Statistical analyses

G*Power 3.1.9 was used to calculate the sample size. The sample size was estimated based on the HOMA-IR after treatment, as this was the primary endpoint. It was assumed that the baseline HOMA-IR was 2.6 (standard deviation (SD), 1.5), and it increased by 32% with rosuvastatin 20 mg and decreased by 34% with telmisartan, but increased by 2% with amlodipine according to previous studies^[11,17]^ It was assumed that the HOMA-IR decreased to 2.54 and increased to 3.48 after a 6-month treatment of telmisartan/rosuvastatin and amlodipine/rosuvastatin, respectively. An estimated sample size of 50 participants per treatment group would be required, considering a dropout rate of 20% and under the statistical conditions as follows: significance level (α), 0.05; power, 0.80 (β = 0.2); and SD (σ), 1.5.

The baseline characteristics were compared between the 2 groups using the χ^2^ test for dichotomous variables or the *t*-test or Mann–Whitney test for continuous variables. The change in variables during the study period was investigated using the paired *t*-test or Wilcoxon signed-rank test according to the data distribution. The differences in the change in variables between the 2 groups were also compared using the *t*-test or Mann–Whitney test. The efficacy endpoints were analyzed using the full analysis set. The multivariate logistic regression models were used for estimating the association between telmisartan use and NODM. Model 1 included age, sex, body mass index (BMI), use of beta-blocker, estimated glomerular filtration rate, and fasting plasma glucose as co-variates. Model 2 included HOMA-IR instead of fasting plasma glucose. A *P* value of <.05 was considered statistically significant. All analyses were performed using the software R version 3.6.0 (R Foundation for Statistical Computing, Vienna, Austria).

## 3. Results

### 3.1. Baseline characteristics of participants

A total of 106 patients provided written consent to participate in the study. However, 6 failed screening based on the exclusion criteria, and one withdrew because of personal reasons. Participants were randomly assigned to the telmisartan-statin group (n=48) and amlodipine-statin group (n=51). The mean age of participants was 60.1 ± 8.8 years, and 78% were men. There were no significant differences in the baseline characteristics between the groups (Table 1).

**Table 1:** Baseline characteristics.

Variables	Telmisartan-statin	Amlodipine-statin	*P* value
	(n = 48)	(n = 51)	
Age, y	61.0 ± 8.8	59.3 ± 8.8	.347
Male, n (%)	39 (81.2)	38 (74.5)	.573
Height, cm	166.8 ± 8.1	166.2 ± 8.4	.712
Weight, cm	71.7 ± 10.3	72.1 ± 12.1	.887
Body mass index, kg/m^2^	25.7 ± 2.9	26.0 ± 3.1	.678
Current smoker, n (%)	11 (22.9)	16 (31.4)	.473
Coronary artery diseases, n (%)	48 (100.0)	51 (100.0)	–
Acute coronary syndrome, n (%)	32 (66.7)	41 (80.4)	.186
Myocardial infarction, n (%)	11 (22.9)	14 (27.5)	.774
Coronary revascularization, n (%)	22 (45.8)	28 (54.9)	.483
Stroke or transient ischemic attack, n (%)	2 (4.2)	2 (3.9)	.999
Peripheral arterial disease or history of peripheral arterial revascularization, n (%)	0 (0.0)	2 (3.9)	.502
Estimated glomerular filtration rate, mL/min/1.73 m^2^	87.8 ± 17.8	85.8 ± 14.7	.542
AST, IU/L	23.8 ± 8.1	25.5 ± 7.8	.291
ALT, IU/L	27.7 ± 14.7	30.0 ± 13.8	.422
Creatine phosphokinase, U/L	117.9 ± 47.6	129.7 ± 69.5	.325
Fasting glucose, mg/dL	111.2 ± 10.2	110.0 ± 10.0	.566
HbA1c, %	6.0 ± 0.3	6.0 ± 0.6	.443
Prior medication, n (%)			
Renin-angiotensin system blockers	27 (56.2)	32 (62.7)	.650
Calcium channel blockers	22 (45.8)	16 (31.4)	.203
Statin	46 (95.8)	50 (98.0)	.957
Combined medication			
Aspirin or clopidogrel	48 (100)	51 (100)	–
Beta-blockade	39 (81.2)	44 (86.3)	.685
Thiazide or thiazide-like diuretics	7 (14.6)	4 (7.8)	.455
Anti-anginal drugs	24 (50.0)	24 (47.1)	.927

Data are presented as mean ± standard deviation or number (%). ALT, alanine aminotransferase; AST, aspartate aminotransaminase; IFG, impaired fasting glucose; IGT, impaired glucose tolerance; HbA1c, hemoglobin A1c.

### 3.2. Changes in BP and lipid profiles

At baseline, systolic and diastolic BP did not differ between the 2 groups (Table S1, Supplementary Digital Content 1, http://links.lww.com/MD/H268 which presents the changes in BP and lipid profiles at baseline and week 24). At week 12, 2 participants in the amlodipine group and 3 in the telmisartan group received an increased dose of the drug because they did not reach the target BP. There was no difference in the systolic and diastolic BP at the end of the study (week 24) between the 2 groups. The changes in systolic/diastolic BP during the study period within each group did not show statistical significance, and there were no group differences in the BP change.

In both groups, the changes in total cholesterol, triglyceride, and LDL-C did not show statistical significance during the study period, and there was no statistical difference between the groups for each change (Table S2, Supplementary Digital Content 2, http://links.lww.com/MD/H269 which shows metabolism profiles change from baseline to week 12), except for a change in HDL-cholesterol levels in the amlodipine-statin group. The percentage change in HDL-cholesterol levels from the baseline to week 24 did not differ between the groups.

### 3.3. Changes in parameters related to glucose metabolism

**Figure 2.**
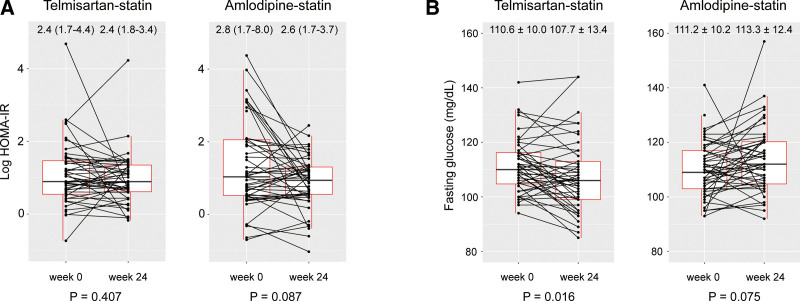
The change of log HOMA-IR (A) and fasting glucose level (B) from week 0 to week 24 in each group. Values on the figure were median (interquartile range) of HOMA-IR or mean ± standard deviation of fasting glucose level. *P* values for Wilcoxon signed-rank test (A) and paired *t* test (B). HOMA-IR = homeostatic model assessment for insulin resistance.

Table 2 presents the changes in parameters related to glucose metabolism during the study period. The values of parameters at baseline were similar between groups. The HOMA-IR at week 24 was not significantly different between the telmisartan-statin and amlodipine-statin groups. Also, the percentage changes in HOMA-IR from weeks 0 to 24 were also not significantly different for both groups (Table 2, Fig. 2A). The telmisartan-statin group maintained the change in HOMA-B from weeks 0 to 24, but it was significantly decreased in the amlodipine-statin group (*P* < .001; Table 2).

**Table 2:** The changes of parameters related to glucose metabolism.

	Telmisartan-statin group	Amlodipine-statin group	*P* value
	(N = 48)	(N = 51)	
HOMA-IR at week 0	2.8 (1.8 to 4.6)	2.8 (1.7 to 7.2)	.800
HOMA-IR at week 24	2.4 (1.8 to 3.8)	2.7 (1.7 to 3.7)	.809
% change	−7.0 (−28.0 to 21.3)	−5.5 (−53.3 to 27.3)	.539
*P* value for Wilcoxon signed-rank test	.407	.087	
HOMA-B at week 0	76.4 (46.8 to 122.9)	86.8 (48.7 to 211.3)	.315
HOMA-B at week 24	74.1 (54.5 to 103.6)	63.6 (45.4 to 102.9)	.252
% change	−6.0 (−23.3 to 26.9)	−19.8 (−52.7 to 3.3)	.040
*P* value for Wilcoxon signed-rank test	.467	<.001	
Fasting glucose at week 0, mg/dL	111.2 ± 10.2	110.0 ± 10.0	.566
Fasting glucose at week 24, mg/dL	107.7 ± 13.4	113.3 ± 12.4	.039
% change	−3.2 ± 8.6	3.8 ± 13.2	.003
*P* value for paired *T* test	.016	.075	
HbA1c at week 0, %	6.0 ± 0.3	6.0 ± 0.6	.443
HbA1c at week 24, %	6.0 ± 0.3	6.0 ± 0.4	.498
% change	0.1 ± 3.2	0.5 ± 4.7	.625
*P* value for paired *T* test	.876	.469	
Insulin at week 0, mIU/L	9.5 (6.4 to 15.9)	10.2 (6.6 to 27.9)	.361
Insulin at week 24, mIU/L	9.2 (7.2 to 13.8)	9.7 (5.9 to 13.8)	.750
% change	−3.6 (−31.6 to 19.5)	−6.8 (−52.2 to 21.1)	.595
*P* value for Wilcoxon signed-rank test	.221	.082	

Data are presented as mean ± standard deviation or median (interquartile range). HbA1c = hemoglobin A1c, HOMA-B = homeostatic model assessment for beta cell function, HOMA-IR = homeostatic model assessment for insulin resistance.

At week 24, the telmisartan-statin group showed lower fasting blood glucose levels than the amlodipine-statin group (*P* = .003; Table 2, Fig. 2B). Additionally, from baseline to week 24, fasting blood glucose levels decreased in the telmisartan-statin group (*P* = .016), but not in the amlodipine-statin group (*P* = .075; Fig. 2B). The percentage change of fasting glucose from baseline to week 24 was also different between the 2 groups (*P* = .040). However, the levels of HbA1c and insulin at week 24 and the change in HbA1c and insulin from weeks 0 to 24 were not significantly different between the 2 groups.

**Figure 3.**
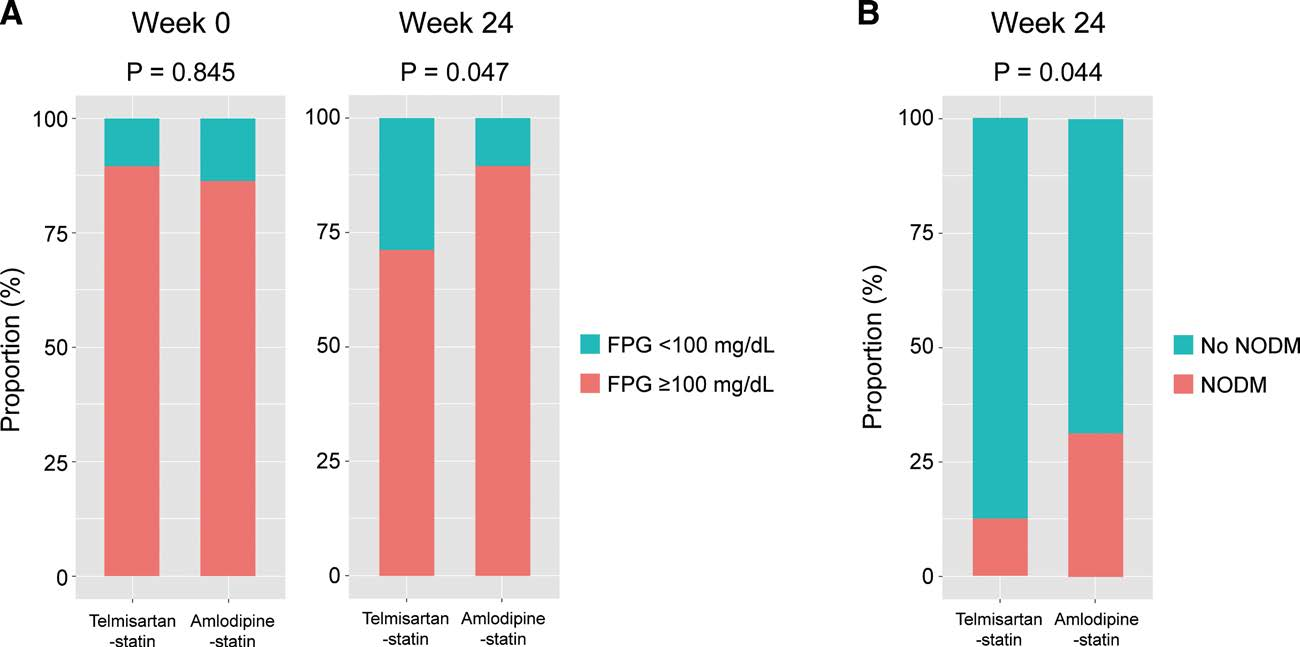
The proportion of participants with fasting plasma glucose ≥100 mg/dL (A) at week 0 and week 24 and new-onset DM (B) at week 24. DM = diabetes mellitus; FPG = fasting plasma glucose; NODM = new-onset DM.

Figure 3 shows the proportion of participants with fasting plasma glucose of ≥100 mg/dL at weeks 0 and 24 and the proportion of NODM. At week 24, the proportion of participants with both fasting plasma glucose of ≥100 mg/dL (71.1% [n=34] vs. 89.6% [n=46], *P* = .047; Fig. 3A) was lower in the telmisartan-statin group than in the amlodipine-statin group. Similarly, the proportion of NODM at week 24 was lower in the telmisartan-statin group than in the amlodipine-statin group (12.5% [n=6] vs. 31.4% [n=16], *P* = .044; Fig. 3B).

In multivariate logistic analyses (Table S3, Supplementary Digital Content 3, http://links.lww.com/MD/H270 which presents independent predictors for developing NODM), the use of telmisartan was an independent protective factor for developing NODM. High baseline fasting plasma glucose was a risk factor for developing NODM, while HOMA-IR was not.

### 3.4. Adverse events during study periods

Of the 99 participants, 16 participants in the telmisartan-statin group and 19 participants in the amlodipine-statin group reported adverse events (Table S4, Supplementary Digital Content 4, http://links.lww.com/MD/H271 which describes adverse events during the study period). There was no statistical difference in overall adverse events between the 2 groups. No serious adverse events occurred. There were 4 cases in which the clinical trial drug likely caused hypotension (n=1), headache (n=1), edema (n=1), and dizziness (n=1) in the amlodipine-statin group. There were 4 cases of facial flushing (n=1), hypotension (n=2), and a rise in creatine phosphokinase (n=1) in the telmisartan-statin group.

## 4. Discussion

The main findings of this study were as follows. First, the addition of amlodipine and telmisartan to patients with IFG who had hypertension and an ASCVD-required statin did not significantly improve HOMA-IR. However, the levels of HOMA-B, an indicator of insulin secretion, were maintained in the telmisartan-statin group, but reduced in the amlodipine-statin group, suggesting the protective effect of telmisartan on beta-cell function. Second, fasting glucose slightly increased at week 24 than at baseline in the amlodipine-statin group, but it decreased significantly during the study period in the telmisartan-statin group, with a lower value at week 24 in the telmisartan-statin group than in the amlodipine-statin group. Third, the telmisartan-statin group showed a lower proportion of participants with fasting plasma glucose of ≥100 mg/dL and a lower rate of NODM at 24 weeks than the amlodipine-statin group. In the multivariate analysis, the use of telmisartan was a significant protective factor for preventing NODM at 24 weeks, demonstrating the beneficial role of telmisartan for ASCVD and IFG patients at risk of DM and requiring high-intensity statins.

Although several studies on the effect of telmisartan on the incidence of DM exist, the distinguishing feature of this study was the focus on patients taking high-intensity statins. In several studies, statins have been shown to increase the risk of DM through various mechanisms. For example, statins can affect pancreatic islet beta-cells and reduce insulin secretion rate.^[18,19]^ Another mechanism is that statins increase insulin resistance. Statin induces the accumulation of fatty acids, which causes a detrimental effect on insulin signaling in muscle cells.^[20]^ Consistent with these results, HOMA-IR showed a significant dose-dependent increase with 10, 20, and 40 mg of rosuvastatin in hyperlipidemia patients with IFG.^[21]^ Studies have shown that patients with several pre-existing risk factors for diabetes are at a higher risk of developing diabetes due to statins.^[3,6]^ In this context, patients at risk of cardiovascular disease could also possibly have a higher risk of developing diabetes due to statins. Although it is strongly recommended not to discontinue statins in patients at a high risk of developing ASCVD because its protective effects on cardiovascular disease outweigh the risk of diabetes,^[1]^ considering the impaired quality of life and the escalation of medical expenses due to DM, the development of NODM must be reduced as well.^[22]^

In ASCVD patients with hypertension and dyslipidemia, the choice of antihypertensive drugs would be a good way to lower the side effects of statins, especially the risk of NODM. Among antihypertensive drugs, which are classified as first-line, beta-blockers and thiazide-like diuretics are known to increase insulin resistance.^[23]^ In contrast, renin-angiotensin-aldosterone system (RAAS) blockades, including ARB, can reduce the risk of NODM through an important role in glucose homeostasis; angiotensin II negatively affects glucose uptake by inhibiting Glucose Transporter Type 4 translocation in the muscle and adipocytes, activates the inflammatory cytokine, and promotes the sympathetic nervous system, thereby increasing blood catecholamine levels and resulting in insulin resistance.^[24,25]^ However, calcium channel blockers, one of the most common antihypertensive drugs, may be associated with a higher incidence of DM than RAAS blockers in hypertensive patients.^[26]^ This trial, COMPROMISE, illustrates that the amlodipine group showed poor glucose metabolism, which may be a detrimental effect caused by calcium channel blocker (CCB). However, in a network meta-analysis comparing the association between antihypertensive drugs and incident DM, ARB or angiotensin-converting enzyme (ACE) inhibitor had the lowest risk and followed by CCB and placebo, beta-blockers, and diuretics in rank order.^[27]^ Compared to placebo, CCB has a lower risk of diabetes, so the detrimental effect of CCB seems to be less likely.^[27]^

Of the many ARBs, telmisartan may be more effective in lowering the risk of diabetes and differs from other ARBs since it is structurally similar to pioglitazone and can activate PPARγ, even at low concentrations.^[10]^ Meta-analysis of 21 randomized clinical trials showed the effect of improving insulin resistance compared to telmisartan and other ARBs.^[28]^ A previous study showed that the combination of rosuvastatin with telmisartan significantly decreased HOMA-IR in patients with IFG compared to irbesartan or olmesartan.^[11]^

In this study, there was no significant difference in HOMA-IR reduction between the treatments. However, 24-week telmisartan treatment preserved the levels of HOMA-B, reduced fasting plasma glucose levels, and resulted in a smaller proportion of participants with fasting plasma glucose of ≥100 mg/dL. This study observed a greater proportion of patients changing from IFG to euglycemic status and a lower incidence of NODM in the group receiving high-intensity statins, than in the group receiving the 24-week amlodipine treatment. Thus, this study was the first to investigate the effects of telmisartan versus amlodipine on glucose homeostasis in hypertensive ASCVD patients with high-dose and high-intensity statin treatment.

The Telmisartan Randomised Assessment Study in ACE Intolerant Subjects With Cardiovascular Disease (TRANSCEND) did not demonstrate any reduction in the risk of NODM compared to placebo in participants at a high risk of cardiovascular disease but free from DM.^[29]^ However, in TRANSCEND, the proportion of participants taking statins with prediabetes was only 57% and 44.8%, respectively. In comparison, the differentiating feature of this study was that all participants had IFG and took a high-intensity statin.

Telmisartan showed a tendency to improve insulin resistance and beta-cell function in patients with insulin resistance compared to placebo.^[13]^ In contrast, the numerical improvement in HOMA-IR was not shown in this study. Generally, telmisartan has a favorable effect on glucose metabolism, but there are also reports indicating that telmisartan has a neutral effect on HOMA-IR.^[30]^ One novelty of this study was that telmisartan showed no changes in HOMA-B compared to amlodipine. HOMA-B is a value that has been validated against complex methods such as the glucose tolerance test and the euglycemic-hyperinsulinemic clamp and has the advantage of being easily calculated with routine laboratory measurements.^[31]^ Low HOMA-B is associated with a high risk of diabetes,^[32]^ and this study suggests that the beneficial effect of telmisartan on glucose metabolism may be due to the mitigation of statin-induced of beta-cell function deterioration. However, since HOMA-B is an indirect measure of beta-cell function only accounting for fasting status, further research is needed.

There were some limitations of this study. First, this study was not double-blinded and not placebo-controlled. Second, because this study targeted patients with clinical ASCVD, most patients had hypertension, and the antihypertensive drug previously administered could have affected the metabolic parameters. Hence, a washout period should be considered after discontinuation of antihypertensive drugs. However, the setting of the washout period was impossible for patients with clinical ASCVD. Third, beta-blockers can affect insulin resistance and glucose metabolism, their effects vary depending on the type of beta-blockers.^[33]^ Since we did not control the type of beta-blockers, we cannot rule out the effects of differences in beta-blockers in use. However, the use rate of beta-blockers between the 2 groups was similar. Fourth, this study did not target statin-naïve patients. Previous studies have shown that the duration, dosage, and type of statin administration can affect diabetes incidence.^[34,35]^ Therefore, the possibility that these factors influenced HOMA-IR cannot be excluded. Fifth, the follow-up duration of the study may have been too short to investigate the change of insulin resistance. The incidence of NODM was less in the telmisartan-statin group in this study, but a longer follow-up period might have helped to show a clear causal relationship between antihypertensive drugs and high-intensity statin-induced insulin resistance. Sixth, this study used HOMA-IR, which is calculated by using fasting insulin and glucose levels, as an index for insulin resistance. There are various indices for insulin resistance, and a more in-depth analysis would have been possible if we had measured postprandial insulin and glucose. Finally, weight changes or lifestyle modifications might affect glucose metabolism even with short-term observation, but these variables were not analyzed.

## 5. Conclusions

In summary, the telmisartan-rosuvastatin treatment combination did not significantly improve insulin resistance, but preserved insulin secretion, improved fasting blood glucose, and reduced the risk of NODM more than the amlodipine-rosuvastatin combination in hypertensive ASCVD patients with IFG.

## Acknowledgments

The authors thank Editage (www.editage.co.kr) for English language editing.

## Author contributions

Conception and design: Chan Joo Lee, Jong-Youn Kim, Byeong-Kuek Kim

Collection and assembly of data: Jung-Hoon Sung, Tae-Soo Kang, Jong-Youn Kim, Byeong-Kuek Kim

Data analysis and interpretation: All authors.

Manuscript writing: Chan Joo Lee, Byeong-Kuek Kim

Final approval of manuscript: All authors.

Accountable for all aspects of the work: All authors.

## Supplementary Material


