# Supplementary material for: Effects of high-intensity statin combined with telmisartan versus amlodipine on glucose metabolism in hypertensive atherosclerotic cardiovascular disease patients with impaired fasting glucose: A randomized multicenter trial
Source: Medicine (Baltimore). 2022 Sep 9;101(36):e30496. doi: 10.1097/MD.0000000000030496 (PMC10980368; doi:10.1097/MD.0000000000030496)
Supplement: Supplementary file 1 [file medi-101-e30496-s001.pdf]

Table S1. Blood pressure and lipid profile changes

Group	Telmisartan-statin group (n = 48)	Amlodipine-statin group (n = 51)	P-value
Office systolic BP at week 0	130.5 ± 12.1	130.6 ± 15.2	0.990
Office systolic BP at week 24	127.1 ± 11.2	129.4 ± 13.0	0.351
Change from week 0 and 24	-2.9 ± 14.3	-1.7 ± 14.1	0.675
P-value for paired T-test	0.177	0.408	
Office diastolic BP at week 0	80.2 ± 9.2	78.8 ± 9.8	0.473
Office diastolic BP at week 24	80.6 ± 7.5	81.7 ± 8.4	0.497
Change from week 0 and 24	0.6 ± 10.2	2.6 ± 9.1	0.329
P-value for paired T-test	0.593	0.057	
Total cholesterol at week 0, mg/dL	141.7 ± 21.7	142.3 ± 22.6	0.908
Total cholesterol at week 24, mg/dL	141.4 ± 29.8	146.6 ± 32.2	0.425
% change	0.1 ± 21.2	3.7 ± 22.6	0.436
P-value for paired T-test	0.565	0.468	
Triglyceride at week 0, mg/dL	145.0 ± 83.1	139.6 ± 61.9	0.718
Triglyceride at week 24, mg/dL	144.5 ± 68.7	141.8 ± 66.3	0.845
% change	8.0 ± 45.9	6.9 ± 41.9	0.906
P-value for paired T-test	0.673	0.968	
HDL-cholesterol at week 0, mg/dL	48.1 ± 10.1	47.2 ± 10.3	0.671
HDL-cholesterol at week 24, mg/dL	47.9 ± 9.8	50.1 ± 12.0	0.331
% change	0.7 ± 14.3	6.5 ± 15.4	0.061
P-value for paired T-test	0.890	0.008	
LDL-cholesterol at week 0, mg/dL	71.6 ± 21.7	71.9 ± 17.8	0.937
LDL-cholesterol at week 24, mg/dL	70.8 ± 23.8	73.5 ± 30.6	0.635
% change	-0.3 ± 33.6	3.7 ± 38.4	0.596
P-value for paired T-test	0.443	0.764	

Data are presented as mean ± standard deviation. BP, blood pressure; HDL, high density lipoprotein; LDL, low density lipoprotein
