# Supplementary material for: Effects of high-intensity statin combined with telmisartan versus amlodipine on glucose metabolism in hypertensive atherosclerotic cardiovascular disease patients with impaired fasting glucose: A randomized multicenter trial
Source: Medicine (Baltimore). 2022 Sep 9;101(36):e30496. doi: 10.1097/MD.0000000000030496 (PMC10980368; doi:10.1097/MD.0000000000030496)
Supplement: Supplementary file 2 [file medi-101-e30496-s002.pdf]

Table S2. Metabolism profile change from baseline to week 12.

	Telmisartan (N=48)	Amlodipine (N=51)	p
HOMA-IR, %	-16.9 (-57.5 to 20.2)	-5.5 (-50.1 to 33.7)	0.617
HOMA-B, %	-18.9 (-42.8 to 43.5)	-17.7 (-42.3 to 22.3)	0.749
Insulin, %	3.9 ± 96.8	5.6 ± 86.4	0.927
Fasting glucose, %	-2.6 ± 7.3	3.6 ± 14.9	0.012
HbA1c, %	0.9 ± 3.2	0.6 ± 5.5	0.749
Total cholesterol, %	1.8 ± 18.8	0.2 ± 17.9	0.664
Triglyceride, %	6.5 ± 43.6	9.4 ± 46.9	0.758
HDL-cholesterol, %	14.4 ± 88.9	4.2 ± 16.0	0.449
LDL-cholesterol, %	2.8 ± 31.2	-3.3 ± 28.3	0.314

HOMA-B, homeostatic model assessment for beta cell function; HOMA-IR, homeostatic model assessment for insulin resistance; HDL, high density lipoprotein; LDL, low density lipoprotein.
