# Supplementary material for: Effects of high-intensity statin combined with telmisartan versus amlodipine on glucose metabolism in hypertensive atherosclerotic cardiovascular disease patients with impaired fasting glucose: A randomized multicenter trial
Source: Medicine (Baltimore). 2022 Sep 9;101(36):e30496. doi: 10.1097/MD.0000000000030496 (PMC10980368; doi:10.1097/MD.0000000000030496)
Supplement: Supplementary file 3 [file medi-101-e30496-s003.pdf]

Table S3. Independent predictors for developing new-onset diabetes mellitus

**Variables**	**OR**	**95% CI**	**P value**
Model 1			
Use of telmisartan (ref=amlodipine)	0.22	0.06-0.68	0.009
Age	0.95	0.88-1.03	0.511
Sex (ref=male)	0.26	0.03-1.21	0.060
BMI at baseline	0.92	0.74-1.14	0.337
FPG at baseline	1.11	1.04-1.18	0.001
Model 2			
Use of telmisartan (ref=amlodipine)	0.28	0.09-0.80	0.023
Age	0.99	0.92-1.06	0.687
Sex (ref=male)	0.24	0.03-1.14	0.110
BMI at baseline	0.94	0.76-1.14	0.527
HOMA-IR at baseline	1.02	0.99-1.06	0.141

BMI, body mass index; CI, confidence interval, FPG, fasting plasma glucose; HOMA-IR, homeostatic model assessment for insulin resistance; OR, odd rate
