# Supplementary material for: Effects of high-intensity statin combined with telmisartan versus amlodipine on glucose metabolism in hypertensive atherosclerotic cardiovascular disease patients with impaired fasting glucose: A randomized multicenter trial
Source: Medicine (Baltimore). 2022 Sep 9;101(36):e30496. doi: 10.1097/MD.0000000000030496 (PMC10980368; doi:10.1097/MD.0000000000030496)
Supplement: Supplementary file 4 [file medi-101-e30496-s004.pdf]

Table S4. Adverse events during the study period.

	**Number of incidence**
Elevation of creatine phosphokinase	1
Foot pain	1
Enteritis	1
Bruise	1
Flushing	1
Mass on chest wall	1
Abdominal pain	1
Elevation of HbA1c	1
Glaucoma	1
Facial palsy	1
Leg swelling	1
Myalgia	2
Upper respiratory infection	2
Dyspnoea	2
Elevation of liver enzyme	4
Dizziness	4
Headache	6
Diabetes	7
Hypertension	6
Hypotension	3
